# EDC-Net for small object detection in UAV imagery using edge-aware dynamic context modeling

**DOI:** 10.1038/s41598-026-58896-y

**Published:** 2026-06-22

**Authors:** Yangyang Gao, Shuxian Liu

**Affiliations:** School of Computer Science and Technology, Xinjiang University, 777 Huarui Street, Shuimogou District, Urumqi, 830017 The Xinjiang Uygur Autonomous Region China

**Keywords:** Unmanned aerial vehicle, Small object detection, Edge feature enhancement, Dynamic context aggregation, Computational biology and bioinformatics, Engineering, Mathematics and computing

## Abstract

To address the challenges of insufficient detection accuracy in UAV aerial imagery, this paper proposes EDC-Net, a small object detection method based on an Edge-Aware Dynamic Context Network. First, an Edge-Aware Enhancement Module (EAEM) is designed to compensate for contour degradation in deep features. By embedding gradient cues into intermediate feature learning and combining them with spatial-context information, EAEM enhances the network’s sensitivity to tiny-object boundaries. Second, we propose a Multi-scale Adaptive Context Module (MACM), which coordinates dynamic non-linear activation, statistical self-attention, and multi-receptive-field refinement to suppress background interference, aggregate global context with linear complexity, and strengthen multi-scale feature representation. Finally, a Dynamic Structural Alignment Head (DSAH) is constructed by introducing Dynamic Snake Convolution into the regression branch, enabling adaptive geometric alignment for irregular targets and improving localization accuracy. Across the VisDrone2019 dataset, experimental results indicate that the proposed approach outperforms the baseline YOLO11s, with gains of 4.5% and 3.0% in mAP@50 and mAP@50:95, respectively, and a 29.8% reduction in the total number of parameters. Additionally, generalization tests on the TinyPerson dataset confirm the capability of the presented method in small object detection tasks.

## Introduction

With the development of object detection algorithms, the application of UAVs in intelligent transportation, power line inspection, agriculture, forest fire prevention and other fields has become more and more extensive. However, limited by the high-altitude viewing angle, UAV aerial images often have problems such as small target size, dense distribution and large scale ratio between targets. Small targets are often compressed or even lost in deep networks, which seriously affects the detection accuracy, so developing small target detection algorithms for UAV platforms has certain research and application value.

Detection algorithms can be divided into two main categories: traditional and Deep Learning-based. Traditional methods find their way to initial success using hand-crafted features and sliding windows, achieving reasonable performance for certain tasks, but the algorithms usually lack robustness, and the complexity is very high. Deep Learning-based algorithms are further categorized into two categories: two-stage and one-stage object detection algorithms. Two-stage object detection algorithms first generate region proposals and subsequently classify the regions and adjust bounding boxes. Although these methods achieve relatively high accuracy, the speed of inference is often limited due to significant computational redundancies. Example algorithms include R-CNN^1^, Fast R-CNN^2^ and Faster R-CNN^3^. One-stage algorithms, such as SSD^4^, RetinaNet^5^, CenterNet^6^, DETR^7^ and the series of YOLO^8^, perform the detection directly on the image without region proposal generation. Their superior inference speed has made them the mainstream algorithms within UAV image processing tasks.

It is also worth noting that the rapid development of deep learning has driven substantial progress in a wide range of related visual perception tasks^9^, where robust feature representation under challenging conditions has emerged as a common research focus. In this context, frequency-domain modeling^10^ and knowledge distillation^11^ have been explored as effective strategies for enhancing feature discriminability and improving model efficiency, respectively. Such cross-task insights provide useful methodological references for addressing the feature representation challenges encountered in UAV small object detection. In a similar spirit, detail-semantic collaborative modeling has also been explored in related visual perception tasks to jointly leverage fine-grained details and semantic context under complex scenes^12^, further underscoring the importance of detail-semantic synergy that is also central to small object detection.

Many researchers have made efforts in recent years to improve the performance of small object detection in UAV images. Ye et al^13^. developed a hybrid CNN-Transformer detection framework that uses a joint reconstruction unit that fuses local convolution and global self-attention in individual feature layers to achieve adaptive enhancement of intra-layer features. Yi et al^14^. proposed the LAR-YOLOv8 algorithm where the local modules are enhanced by a dual-branch architecture attention mechanism based on YOLOv8. Li et al^15^. designed the Enhanced Scale Sequence Fusion (ESSF) module, which combines SSFF, TFE, and the P2 detection layer to strengthen the multi-scale feature fusion capability of the network. In order to address the problem of computational efficiency, Xuan et al^16^. proposed LAM-YOLOv11 with the LA-C3K2 depthwise separable convolution.

Although these methods have improved detection accuracy to a certain extent, issues such as the small image area and mutual occlusion of small objects, and scene variability in complex aerial environments continue to induce missed detections and false positives. There are three main reasons for these challenges. First, the pixel occupancy of targets in UAV images is very low, and the deeper the network, the more elemental edge and contour information of small objects is lost by repeated downsampling, resulting in fuzzy localization boundaries. Second, small objects are severely affected by background noise and textures that resemble the desired targets. Third, traditional Convolutional Neural Networks rely on static convolution kernels and lack adaptive feature extraction capability.

Existing methods have advanced UAV small-object detection from several directions, such as multi-scale feature fusion, attention-based enhancement, and focusing on difficult regions. However, many existing methods tend to emphasize one specific aspect of the detection pipeline. In aerial scenes, the three main difficulties, namely boundary degradation, background interference, and geometric mismatch, are interrelated and affect different stages of detection, so improving only one aspect may be insufficient to fully address the challenges of UAV small-object detection. This motivates a coordinated stage-wise design organized around these three interrelated difficulties, in which edge enhancement, context modeling, and localization refinement are performed at the backbone, the deep-feature stage, and the detection head, respectively, so that these processes can reinforce one another within a unified detection framework.

To address these issues, we present EDC-Net, an enhanced approach built upon YOLO11, specifically designed for UAV aerial scenarios. The main contributions are listed below:

1. We design an Edge-Aware Enhancement Module (EAEM) to compensate for the contour degradation of tiny objects in deep features. EAEM reformulates the classical Sobel operator as a fixed gradient prior implemented via differentiable group convolution, and pairs it with a parallel spatial-context branch to form a dual-branch structure that fuses explicit edge cues with spatial details. By replacing the original C3k2 modules at multiple backbone stages, EAEM re-injects high-frequency contour information that would otherwise be eroded by repeated downsampling.
2. We propose a Multi-scale Adaptive Context Module (MACM) with a two-stage progressive architecture, in which DyT, TSSA, and Mona play complementary roles. DyT adaptively modulates feature responses to suppress background noise, TSSA aggregates global contextual information with linear complexity, and Mona refines local multi-scale details through multi-receptive-field depth-wise convolution branches.
3. We construct a Dynamic Structural Alignment Head (DSAH), in which Dynamic Snake Convolution is introduced into the regression branch to improve receptive-field alignment for irregular aerial targets, while maintaining a lightweight classification branch. This design enhances localization accuracy without introducing unnecessary computational burden.

## Related work

### Overview of YOLO11

YOLO (You Only Look Once) is a ground-breaking object detection algorithm proposed by Redmon et al^17^.. As a one-stage detector, it achieves fast inference speed while maintaining good detection accuracy. In 2024, Ultralytics released YOLO11^18^, a new detection model based on YOLOv8. This model provides a better balance among speed, accuracy and load. YOLO11 retains the overall structure of YOLOv8, with core innovations mainly concentrated in three modules: C3k2, C2PSA and the detection head. The C3k2 module adds variable kernel sizes and a dual-branch parallel structure to the original CSP-Bottleneck, allowing the network to capture multi-scale contextual information more flexibly. The C2PSA module combines parallel multi-scale convolutions with spatial attention to dynamically enhance channel-wise feature responses. Regarding the detection head, the two standard convolutional layers inside the classification branch are substituted with depthwise separable convolutions. This reduces the computational cost without sacrificing detection precision.

### Small object detection

The MS COCO dataset^19^ provides the most widely accepted standard for defining small objects, which defines small objects as those whose resolutions are below 32 *×*32 pixels. Those objects have very few effective pixels and thus only contain very limited feature information, making them easily affected by complex backgrounds. This makes it quite difficult for detection models to locate them accurately. Therefore, solving the problems of insufficient detailed information and weak anti-interference ability of small objects has become an important research focus in this field. Current mainstream research methods mainly include contextual learning, multi-scale learning, attention mechanisms, and region focusing. Amudhan et al^20^. constructed shortcut connections between low-level and high-level features, which greatly boosts the detection performance for small objects in aerial imagery. Zhang et al^21^. introduced CFANet, which adopts the Cross-layer Feature Aggregation (CFA) block to narrow the semantic discrepancy during feature fusion. Leng et al^22^. started from the fact that detection difficulty varies in different regions. They guided the network to focus on areas with difficult targets, and first introduced regional context to help detect these challenging regions, thus improving detection performance. In addition, recent studies on aerial and remote sensing imagery have explored task-specific feature modeling strategies for complex scenes. Xie et al^23^. proposed LBA-MCNet for salient object detection in remote sensing images, which jointly models localization, balance, and affinity to distinguish target regions from cluttered backgrounds. For aerial landslide extraction, Xie et al^24^. developed a context-association framework that explicitly models multi-scale context association to improve feature discrimination under scale variation and background interference.

### Attention mechanisms

As an important technology in deep learning, attention mechanisms are largely inspired by the human visual system. They guide models to focus on the most relevant features during information processing, giving neural networks the perceptual adaptability needed for specific visual tasks. Among classic methods, Woo et al^25^. introduced the Convolutional Block Attention Module (CBAM), which optimizes feature maps across different dimensions, thereby improving feature representation capability. Yang et al^26^. introduced the SimAM mechanism, which adopts an energy-based function to compute 3D attention weights and enables the model to capture useful local features. To handle multiple scales, Ouyang et al^27^. designed the Efficient Multi-scale Attention (EMA) module. It obtains spatial information at various scales through a parallel sub-network structure for effective feature representation. Huang et al^28^. designed the Channel Prior Convolutional Attention (CPCA) mechanism. It uses depthwise convolutions at multiple scales to retain channel priors and capture spatial correlations. Beyond classic attention designs, attention mechanisms have also been applied to fine-grained detection tasks under complex backgrounds. Song et al^29^. proposed a visual attention-guided framework for concrete crack detection, which combines deformable convolutions with spatial-channel attention to enhance subtle target features against strong background interference.

## Methodology

### EDC-Net

**Fig. 1:**
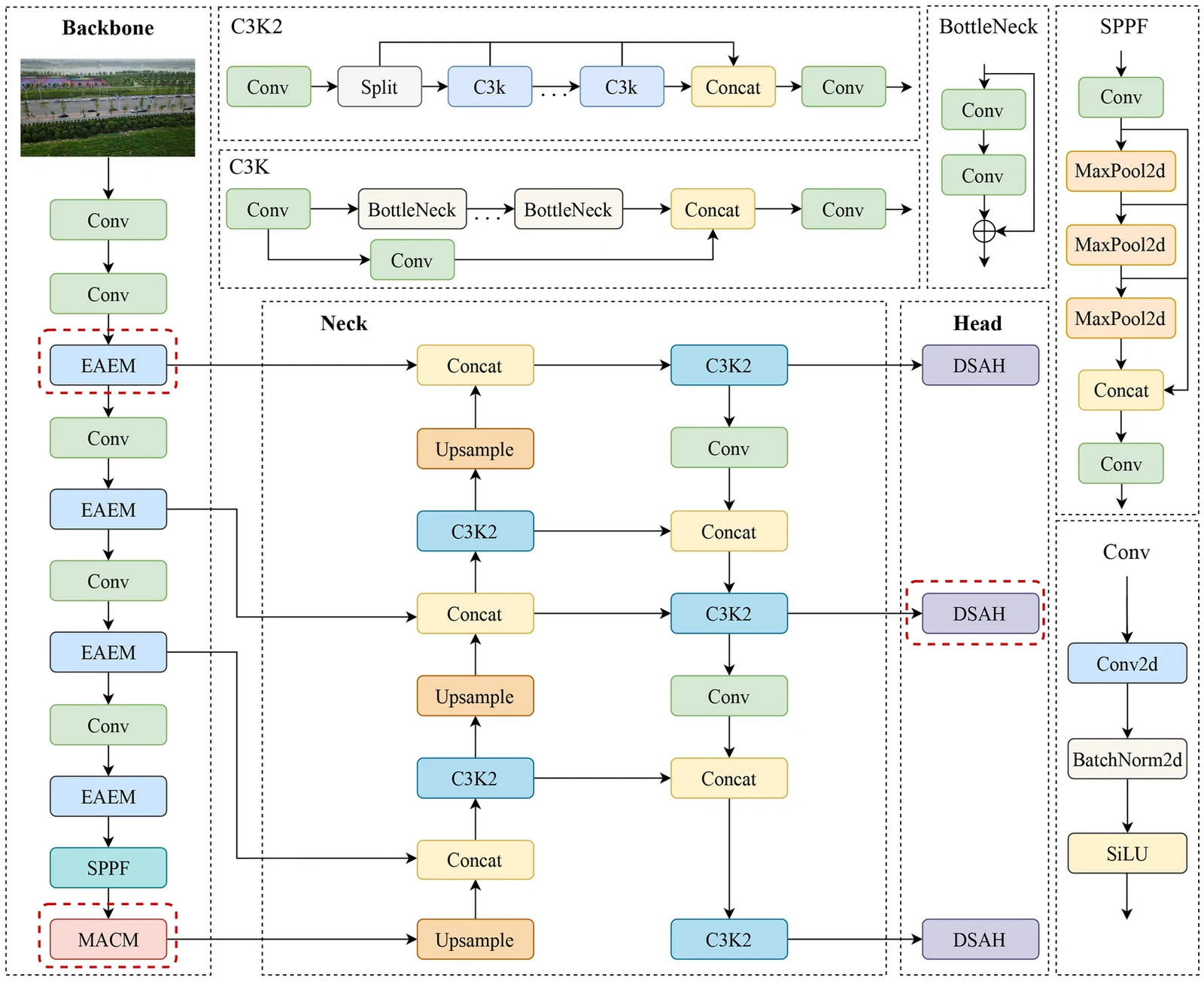
The network structure of EDC-Net.

In order to improve the precision for UAV aerial images, we propose EDC-Net, an improved YOLO11s model, the architecture of which is shown in Fig. 1. To alleviate the loss of texture details in deep features and improve edge extraction for small objects, we design the EAEM module to replace the original C3k2 structure in the backbone network. We then propose the MACM module. By replacing the C2PSA, we achieve an enhanced multi-scale feature representation and simultaneously suppress background noise. Finally, we construct the DSAH to replace the original detection head. By using Dynamic Snake Convolution to adaptively perceive target morphology, this head effectively improves the localization accuracy of the model.

The design of EDC-Net follows a stage-wise principle: each module is placed at the network stage where the corresponding difficulty in UAV small-object detection is most prominent. At the shallow-to-middle backbone stages, where the contour cues of tiny objects are vulnerable to repeated downsampling, EAEM injects an explicit gradient prior to preserve high-frequency boundary information before it is substantially weakened. The edge-enriched features are then processed by MACM in the deeper layers, where background noise and target-like textures are more pronounced. In MACM, dynamic nonlinear modulation suppresses abnormal background responses, statistical self-attention aggregates global context with linear complexity, and multi-receptive-field refinement consolidates multi-scale representations, allowing target-related features to be distinguished from cluttered aerial backgrounds. The refined features are finally delivered to DSAH, in which topology-constrained deformable sampling aligns the receptive field of the regression branch with the irregular geometry of small targets to improve localization accuracy. Through this arrangement, the three modules perform boundary recovery in shallow features, context modeling in deep features, and geometric alignment at the detection head, with each stage operating on the representation refined by the preceding one. In this way, edge enhancement, context aggregation, and localization refinement reinforce one another along the feature hierarchy within a unified detection pipeline.

### Edge-aware enhancement module

**Fig. 2:**
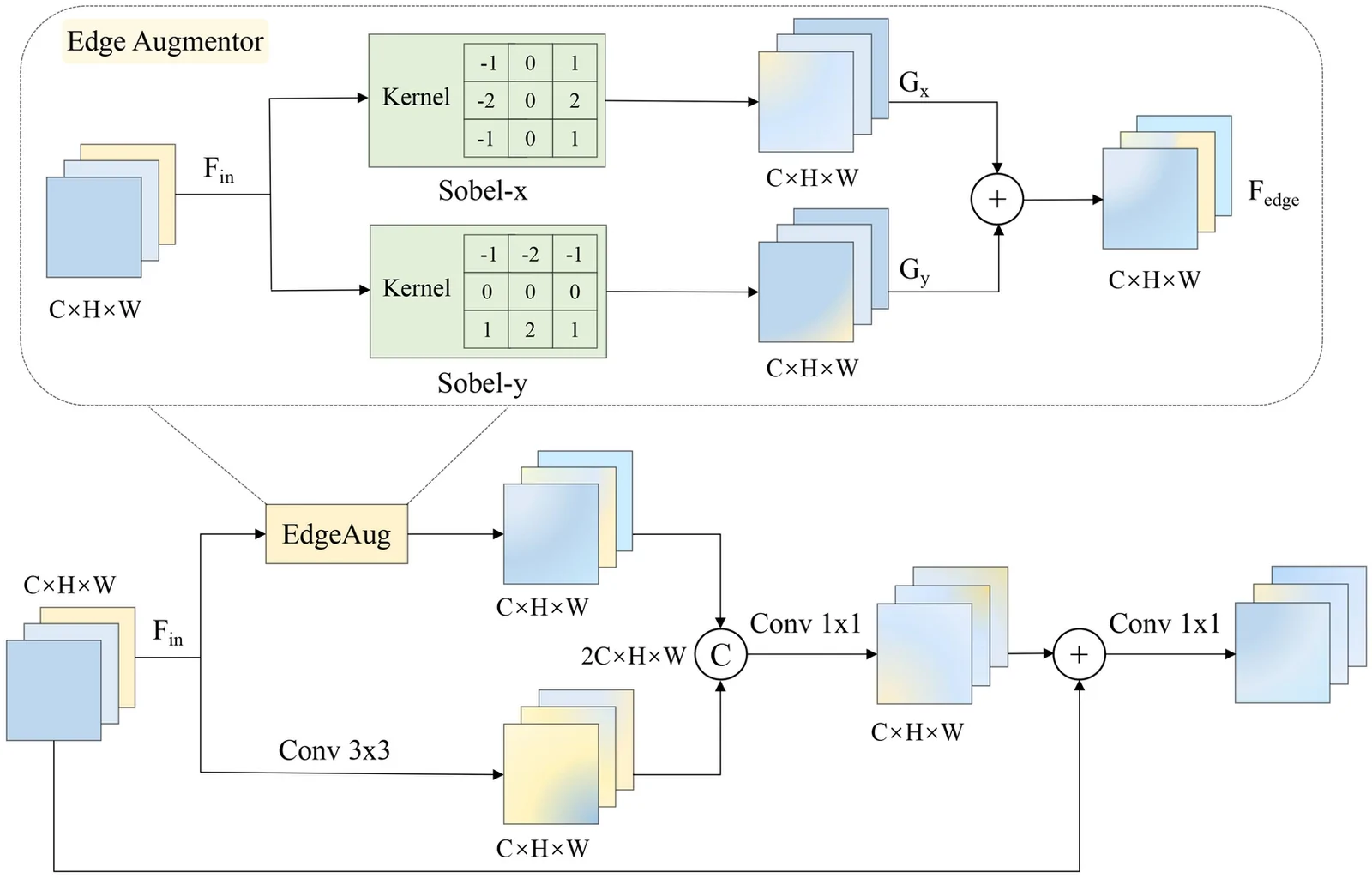
The structure of the Edge-Aware Enhancement Module (EAEM).

To enhance the network’s feature extraction capability, YOLO11 builds upon YOLOv8 by introducing the C3k2 module, which supports custom kernel sizes. This design enables the feature extraction module to adapt more effectively to image features across varying scales. However, in real UAV aerial scenes, targets usually take up very few pixels and are often found in complex backgrounds or heavy occlusions. In these situations, traditional feature extraction modules cannot effectively capture the inner texture details of targets. These details are easily covered by background noise, which leads to blurry boundaries and even the loss of key features. To address the above issues, this paper proposes an Edge-Aware Enhancement Module (EAEM) to rebuild the backbone network by replacing the original C3k2 module. Since EAEM is hierarchically embedded at multiple stages of the backbone, edge cues can be extracted at different spatial resolutions, which helps alleviate the scale sensitivity of the fixed 3*×*3 gradient kernel. As shown in Fig. 2, the EAEM introduces explicit edge prior knowledge to build a dual-branch parallel structure. This structure combines edge features with spatial details, making the network more sensitive to the contours of tiny objects in complex backgrounds. Rather than using the Sobel operator as an independent image-level preprocessing step, EAEM embeds this classical gradient prior into intermediate feature processing and integrates it with a learnable spatial-context branch for branch-level feature fusion.

Specifically, the input feature map is passed to the edge-aware branch and the spatial context branch, which perform edge feature extraction and spatial context preservation, respectively. In the edge-aware branch, the Edge Augmentor (EdgeAug) designed in this paper is used to reinforce target contours. This module includes *3 × 3* Sobel convolution kernels in both horizontal (Sobel-x) and vertical (Sobel-y) directions, efficiently implementing the Sobel operator with a set of differentiable 3D group convolutional layers to compute gradient responses from intermediate feature maps. In this way, the fixed gradient prior is embedded into the edge-aware branch and can be further fused with learnable spatial-context features. The convolution kernels of the Sobel operator in the horizontal and vertical directions are defined in Equation (1):

1 $$\begin{aligned} K_{sobel-x} = \begin{bmatrix} -1 & 0 & 1 \\ -2 & 0 & 2 \\ -1 & 0 & 1 \end{bmatrix}, \quad K_{sobel-y} = \begin{bmatrix} -1 & -2 & -1 \\ 0 & 0 & 0 \\ 1 & 2 & 1 \end{bmatrix}, \end{aligned}$$

The input feature map is denoted as $F_{in} \in \mathbb {R}^{C \times H \times W}$, where *C* is the number of channels, and *H × W* is the spatial size of the feature map. The horizontal gradient map $G_x$ and vertical gradient map $G_y$ are obtained through 3D group convolution operations. These two maps are fused by the $L_1$ norm to generate the final edge feature map $F_{edge}$. The calculation process is shown in Equations (2), (3), and (4):

2 $$\begin{aligned} & G_x = F_{in} * K_{sobel-x}, \end{aligned}$$

3 $$\begin{aligned} & G_y = F_{in} * K_{sobel-y}, \end{aligned}$$

4 $$\begin{aligned} & F_{edge} = |G_x| + |G_y|, \end{aligned}$$

Here, * denotes the convolution operation. This process allows the network to acquire clear information about edge structure early in training, and effectively enhances the perception of the contours of small objects. Notably, the Sobel operator is applied to deep feature maps rather than raw RGB pixels. These feature maps have been progressively transformed by preceding convolutional layers, which reduces the dominance of low-level texture noise in the extracted edge responses

Meanwhile, the spatial context branch adopts a standard *3 × 3* convolutional layer to process the input $F_{in}$. This operation preserves the spatial distribution information and low-frequency texture details, obtaining the spatial feature map $F_{spatial}$. To achieve effective complementarity between edge priors and spatial context information, EAEM concatenates the outputs from these two branches along the channel dimension. Feature fusion and channel dimensionality reduction are then performed by a *1 × 1* convolutional layer. By integrating spatial context with gradient cues, this fusion mechanism helps suppress isolated noise responses and irrelevant background edges that lack contextual support, thereby improving the discriminative quality of the fused edge features. In order to facilitate the propagation of features through deep networks and mitigate the vanishing gradient problem, the module introduces a residual connection, adding the fused features to the original input to obtain the final output feature map $F_{out}$.

The dual-branch structure of EAEM greatly improves the discriminability of shallow features in aerial scenes. Unlike traditional CNNs that learn features implicitly, the edge-aware branch reformulates the classical Sobel operator as differentiable group convolution to extract gradient responses from intermediate feature maps. This effectively alleviates the edge blurring problem caused by insufficient pixels and repeated downsampling. The edge-aware branch is sensitive to changes in lighting and contrast, while the spatial context branch maintains the semantic consistency and local spatial details of features. The complementarity of the two branches makes the model more robust against complex background interference.

### Multi-scale adaptive context module

**Fig. 3:**
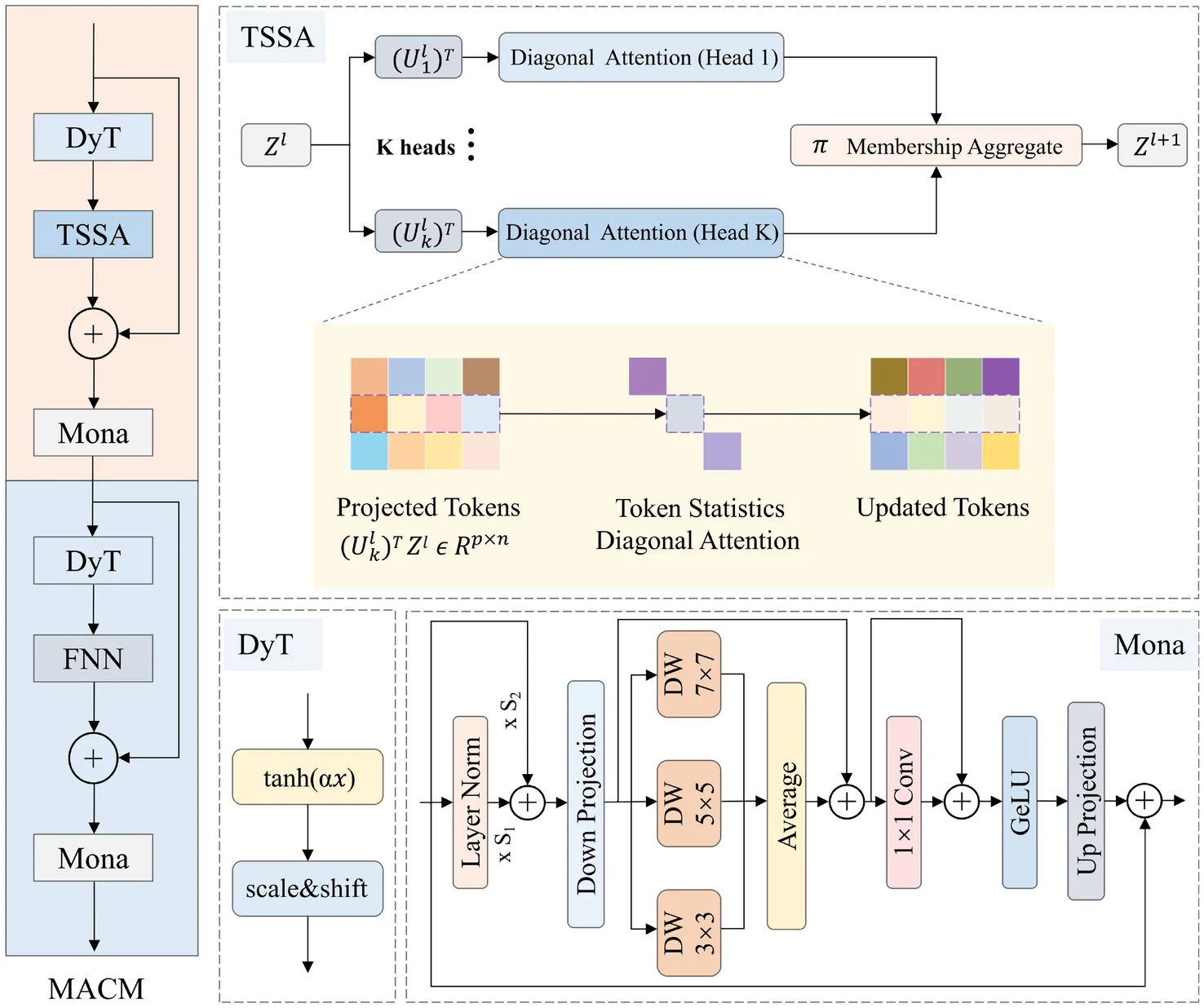
The structure of the Multi-scale Adaptive Context Module (MACM).

In the YOLO11 architecture, the C2PSA module is introduced to better capture global feature dependencies. However, its limitations in computational efficiency and feature representation become evident when dealing with challenging UAV aerial images. First, the self-attention mechanism in C2PSA relies on pixel similarity calculations. This inevitably introduces quadratic computational complexity and hinders the real-time application of the model. Second, C2PSA lacks an effective suppression mechanism for high-frequency background noise. It struggles to differentiate between background noise and small objects. This leads to the excessive reinforcement of background textures. Finally, its existing approach to feature interaction still shows some weaknesses in multiscale representation capabilities. As a result, adapting to the significant scale variations in aerial scenes is challenging. To address these issues, this paper proposes a Multi-scale Adaptive Context Module (MACM), in which DyT, TSSA, and Mona respectively handle background noise interference, the quadratic complexity of global context modeling, and limited multi-scale perception. As shown in Fig. 3, MACM adopts a two-stage progressive enhancement architecture.

Specifically, the MACM pipeline consists of two sequential stages. First, to address the challenge of severe background noise interference in aerial imagery, a Dynamic Tanh (DyT) mechanism^30^ is introduced at the input of each stage. Unlike conventional normalization methods, DyT dynamically amplifies effective feature signals through a learnable scaling factor that adaptively modulates the nonlinear activation response. This design enhances target-related features while suppressing abnormal responses caused by background noise, without introducing the additional statistical computation of normalization layers. The formula of DyT is shown in Equation (5):

5 $$\begin{aligned} \text {DyT}(x) = \gamma \cdot \tanh (\alpha \cdot x) + \beta , \end{aligned}$$

where *α* is a learnable scalar that dynamically scales the input range, controlling the sensitive region of the tanh. *β* and *γ* are learnable vectors that shift and scale across the channels, simulating the affine transformation in a normalization layer.

Second, to achieve efficient global context aggregation, the first stage introduces a novel Token Statistical Self-Attention (TSSA) mechanism^31^. TSSA provides a lightweight and robust method for attention computation by statistically modeling token feature distributions. Its main advantages lie in its linear computational complexity, which reduces the computational burden compared with conventional quadratic-complexity self-attention. Instead of relying on dot products to calculate inter-token dependencies, TSSA uses statistical metrics such as mean and covariance to model attention weights within a more stable statistical space, making it less sensitive to noisy local responses and more suitable for densely distributed, low-contrast small objects with limited pixel-level features in UAV images. The TSSA operator is defined in Equation (6):

6 $$\begin{aligned} \text {TSSA}(Z \mid \{U_k\}_{k=1}^K) \doteq -\frac{\tau }{n} \sum _{k=1}^{K} U_k D(Z, \pi _k \mid U_k) U_k^\top Z \text {Diag}(\pi _k), \end{aligned}$$

where *Z* is a token feature matrix, $\{U_k\}$ is a set of projection matrices, *τ* is the gradient descent step size, $\pi _k$ is a token assignment probability vector, and *D(· )* is a function describing statistical characteristics. By adopting low-rank projections and statistical computations, TSSA abandons the traditional pairwise similarity calculation. This significantly lowers the computational cost while still maintaining effective feature interactions.

Finally, to handle the large scale variations of objects and the frequent feature loss of tiny objects in UAV aerial images, a Multiple Cognitive Vision Adapter (Mona)^32^ is applied at the end of both stages. Mona contains two key processing steps. First, it uses a learnable normalization mechanism to dynamically adjust feature distributions, which suppresses background noise and strengthens task-related features. Second, it realizes multi-scale perception with three parallel depthwise convolution branches. These branches use *3 × 3*, *5 × 5*, and *7 × 7* kernels to capture features with different receptive fields. This allows the network to capture both the local textures of tiny objects and the structural contours of large objects, enabling cross-scale synergistic feature modeling. Then, the multi-scale features are combined by a *1 × 1* convolution and further fused through a residual connection. The computation process is given in Equation (7):

7 $$\begin{aligned} f_{multi} = \text {Conv}_{1 \times 1} \left( \frac{1}{3} \sum _{k \in \{3, 5, 7\}} \text {DWConv}_{k \times k}(x_{norm}) \right) . \end{aligned}$$

Unlike conventional multi-scale convolution modules that mainly aggregate local features through parallel convolutional branches, Mona is applied after DyT-based feature enhancement and TSSA-based global context modeling. Therefore, its multi-scale branches further refine the feature representation rather than independently aggregating multi-scale cues from unprocessed features.

The overall two-stage process is formulated in Equations (8) and (9):

8 $$\begin{aligned} & X_{mid} = M_{ona} \left( X_{in} + A_{TSSA}(J_{dyn}^1(X_{in})) \right) , \end{aligned}$$

9 $$\begin{aligned} & Y_{out} = M_{ona} \left( X_{mid} + F_{FFN}(J_{dyn}^2(X_{mid})) \right) , \end{aligned}$$

Here, $J_{dyn}$, ${A}_{TSSA}$, and ${M}_{ona}$ denote the dynamic nonlinear activation transformation, global statistical attention operation, and multi-scale perception operation, respectively.

Overall, MACM progressively enhances feature representation through noise suppression, global context modeling, and multi-scale perception. DyT is deliberately placed at the input of each stage to deliver a cleaner feature distribution to the subsequent operators, so that TSSA can perform statistical attention computation under a more stable feature space without being disturbed by high-frequency background responses. TSSA then aggregates long-range dependencies with linear complexity, providing globally-aware representations for the subsequent multi-scale refinement. Based on the globally aggregated features, Mona further introduces multi-receptive-field depth-wise convolution branches to recover local texture details and strengthen scale-aware representations. Through this sequential synergy of noise suppression, linear-complexity global modeling, and multi-scale local refinement, MACM enhances the feature discriminability of small objects and improves both detection accuracy and robustness in complex aerial scenes.

### Dynamic structural alignment head

**Fig. 4:**
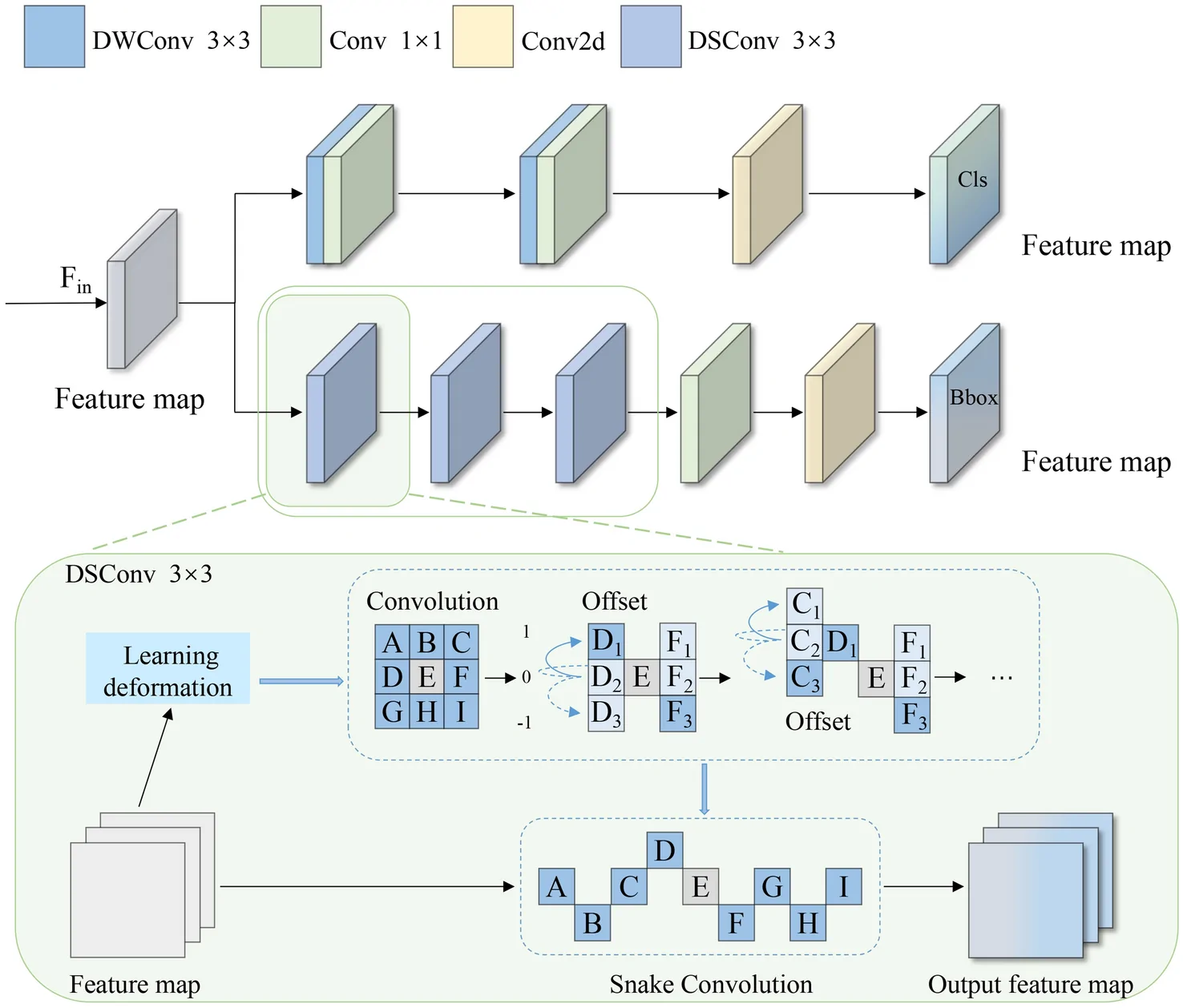
The structure of the Dynamic Structural Alignment Head (DSAH).

Although YOLO11 adopts a decoupled head to enhance task independence, its feature extraction still relies on standard 3*×*3 convolutions. This fixed grid sampling mechanism is inherently more suitable for objects with regular geometries. It exhibits two limitations in UAV aerial scenarios: on the one hand, it struggles to adapt to the irregularly shaped objects commonly found in aerial images; on the other hand, when a target occupies only a handful of pixels, the rigid 3*×*3 grid may introduce irrelevant background responses into the receptive field, which is particularly harmful for tiny objects with low signal-to-noise ratios. Aiming at the above issues, this paper proposes a Dynamic Structural Alignment Head (DSAH). As illustrated in Fig. 4, the DSAH maintains a lightweight classification branch. Meanwhile, it introduces Dynamic Snake Convolution (DSConv)^33^ into the regression branch to enhance the perception capability of the geometric structures of objects. This selective placement concentrates the topology-constrained deformable sampling of DSConv on the localization-sensitive regression branch, thereby alleviating the receptive-field mismatch caused by rigid grid sampling.

Specifically, the regression branch stacks three Dynamic Snake Convolution modules and a *1 × 1* convolutional layer, instead of the pair of *3 × 3* convolutional layers in the initial detection head. Ultimately, a standard convolutional layer computes the final output. The core of DSConv lies in its topology-constrained deformable sampling strategy. Unlike standard deformable convolutions that independently predict the offset for each sampling point, DSConv considers the continuity among the coordinates of the sampling points. Let $K_i = (x_i, y_i)$ denote the center coordinates of a standard *3 × 3* convolutional kernel. Taking the *x*-axis direction as an example, the specific position of each grid within the convolutional kernel *K* is expressed as $K_{i \pm c} = (x_{i \pm c}, y_{i \pm c})$, for $c = \{0, 1, 2, 3, 4\}$. Here, *c* represents the horizontal distance from the center grid. Starting from the center position $K_i$, the selection of each grid position $K_{i \pm c}$ within the kernel is a cumulative process. Specifically, $K_{i \pm c}$ is located by adding an offset $\Delta = \{\delta \mid \delta \in [-1, 1]\}$ to the position of the preceding grid $K_{i \pm c-1}$. Since each offset is bounded and applied cumulatively, the resulting sampling trajectory preserves local structural continuity. This helps prevent the receptive field from drifting into background regions, which is particularly critical for tiny targets in complex aerial scenes.

The variation along the *x*-axis is formulated in Equation (10):

10 $$\begin{aligned} K_{i \pm c} = {\left\{ \begin{array}{ll} (x_{i+c}, y_{i+c}) = (x_i + c, y_i + \sum _{i}^{i+c} \Delta y) \\ (x_{i-c}, y_{i-c}) = (x_i - c, y_i + \sum _{i-c}^{i} \Delta y) \end{array}\right. }, \end{aligned}$$

The variation along the *y*-axis is formulated in Equation (11):

11 $$\begin{aligned} K_{j \pm c} = {\left\{ \begin{array}{ll} (x_{j+c}, y_{j+c}) = (x_j + \sum _{j}^{j+c} \Delta x, y_j + c) \\ (x_{j-c}, y_{j-c}) = (x_j + \sum _{j-c}^{j} \Delta x, y_j - c) \end{array}\right. }, \end{aligned}$$

Since the deformed convolutional kernel may land on fractional coordinates, bilinear interpolation is employed to estimate the corresponding pixel values. The specific formulation is shown in Equation (12):

12 $$\begin{aligned} K = \sum \nolimits _{K'} B(K', K) \cdot K', \end{aligned}$$

Here, *K* is the current fractional coordinate to be calculated, *K'* is the integral coordinate on the feature map, and *B(K', K)* is the bilinear interpolation kernel that can be separated into two one-dimensional kernels as in Equation (13):

13 $$\begin{aligned} B(K, K') = b(K_x, K'_x) \cdot b(K_y, K'_y). \end{aligned}$$

Although DSConv was originally proposed for tubular or continuous structure segmentation, the continuity-constrained deformable sampling at its core is a general feature-sampling mechanism that is suitable for geometry-aware localization. As defined in Equations (10)–(11), the sampling positions are generated by accumulating bounded offsets, so that adjacent sampling points remain spatially coherent and the receptive field can deform along a connected trajectory across the feature map. For bounding box regression, the accuracy of the predicted boundaries depends on whether the aggregated features are dominated by target-related responses. When a target occupies only a few pixels, a standard 3*×*3 grid covers a fixed square region in which surrounding background responses may weaken the reliability of the regressed coordinates. By contrast, the continuity-constrained sampling of DSConv enables the receptive field to better adapt to the compact distribution of effective pixels and the irregular shapes of aerial targets, so that the aggregated features are more informative for boundary localization. In this way, DSConv equips the regression branch with geometry-aware feature alignment and improves its robustness against background interference during localization.

In summary, DSAH utilizes DSConv to endow the regression branch with strong geometric perception. Beyond fitting explicit object shapes, this snake-like sampling mechanism enables the convolutional kernel to adaptively focus on the compact cluster of effective pixels belonging to a tiny target, thereby reducing background interference within the receptive field. Moreover, by recovering deformed sampling positions through bilinear interpolation, DSConv enables finer-grained feature alignment than standard grid-based convolution, which is particularly beneficial for tiny objects whose localization accuracy is highly sensitive to pixel-level misalignment. Together, these advantages alleviate the receptive-field mismatch of traditional convolutions for tiny and irregularly shaped objects, thereby improving their localization accuracy in complex aerial scenes.

## Experiments and result analysis

### Datasets

This study employs two representative public datasets: VisDrone2019^34^ and TinyPerson^35^.

The VisDrone2019 dataset contains 10,209 images, split into 6,471 for training, 548 for validation, and 1,610 for testing. It has ten categories: pedestrian, person, bicycle, car, van, truck, tricycle, awning-tricycle, bus, and motor. With a large number of targets, highly dense small objects, and severe occlusions, this dataset presents a great challenge for small object detection.

**Fig. 5:**
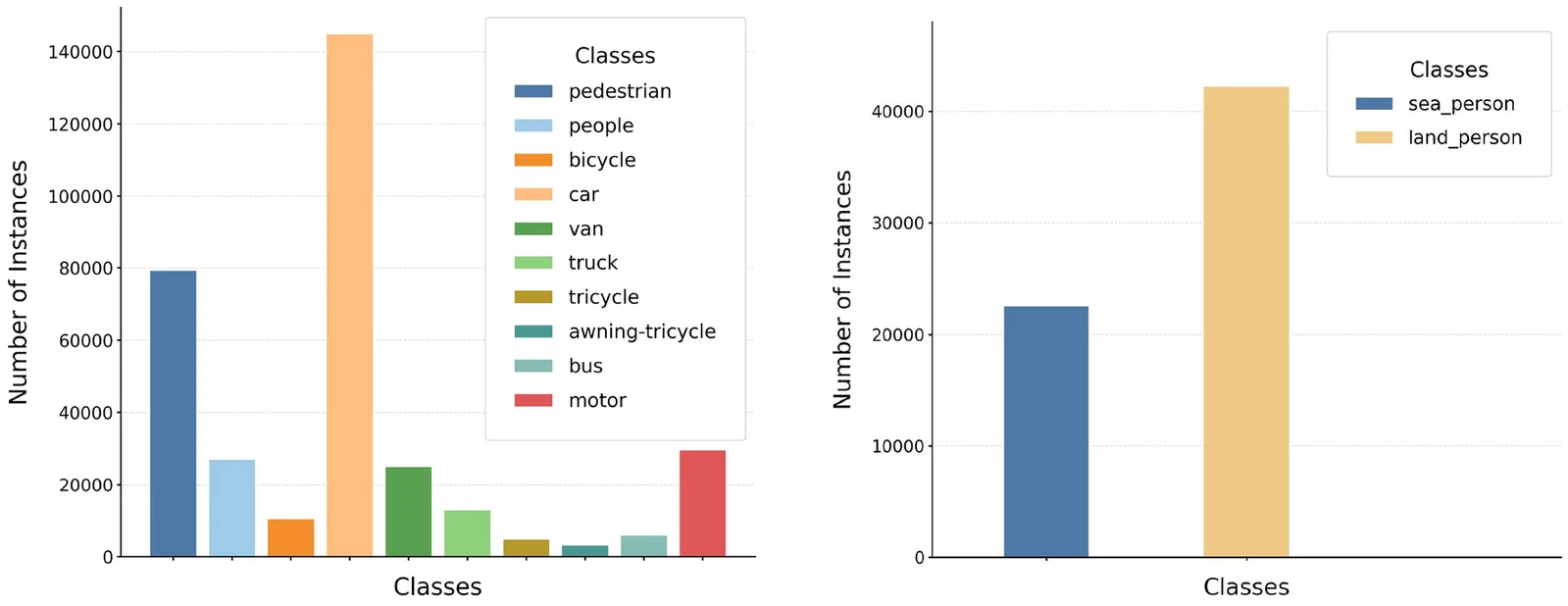
Number of class samples in the VisDrone2019 and TinyPerson datasets.

The TinyPerson dataset contains 1,610 images, which are divided into 796 training samples, 219 validation samples and 595 test samples. It focuses on long-distance person detection and has two categories: sea person and land person. Fig. 5 illustrates the number of class samples for both datasets.

### Evaluation metrics

To quantitatively evaluate the detection performance of the proposed model, this study uses several metrics, including Precision (P), Recall (R), mean Average Precision (mAP), number of parameters (Parameters), model size (Model Size), FPS, GFLOPs, and latency.

Precision represents the proportion of actual positive samples among all samples predicted as positive. Recall denotes the proportion of genuinely positive instances that are successfully identified by the model. Mean Average Precision (mAP) serves as the primary metric for assessing the comprehensive performance of object detection models. mAP@50 is the average precision at an IoU threshold of 0.5, while mAP@50:95 is the average precision at IoU thresholds from 0.5 to 0.95. The number of parameters and model size measure the complexity and storage requirements of the model.

The specific formulas are given in Equations (14) to (17):

14 $$\begin{aligned} & P = \frac{TP}{TP + FP} \end{aligned}$$

15 $$\begin{aligned} & R = \frac{TP}{TP + FN} \end{aligned}$$

16 $$\begin{aligned} & \textrm{AP} = \int _{0}^{1} P(R) dR \end{aligned}$$

17 $$\begin{aligned} & \textrm{mAP} = \frac{1}{N} \sum _{i=1}^{N} \textrm{AP}_i \end{aligned}$$

TP represents the actual positive samples identified as positive. FP represents the actual negative samples identified as positive. FN represents the actual positive samples identified as negative. N is the total number of categories, *i* is the *i*-th category, AP is the average precision of a single category, and mAP is the mean average precision of all categories.

### Experimental environment and parameter settings

To fairly evaluate the proposed model and its improvements, all experiments use the same hardware and software settings, as detailed in Table 1. The training hyperparameters are listed in Table 2.

**Table 1:** Experimental environment.

Parameters	Configuration
GPU	NVIDIA GeForce RTX 3090
CPU	Intel(R) Xeon(R) Platinum 8352V
Accelerated environment	CUDA 11.8
Deep learning framework	PyTorch 2.0.0
Programming Language	Python 3.8.10

**Table 2:** Parameter settings.

Parameter	Value
Epoch	250
Optimizer	SGD
Input images size	*640 × 640*
Batch size	16
Patience	100
Worker	4
Learning rate	0.01
Weight decay	0.0005

To ensure a fair comparison, all comparative methods and the proposed EDC-Net were trained from scratch without using pretrained weights. The goal of this setting is to evaluate all architectures under identical training and data conditions, so that the observed performance differences can be attributed mainly to the network design itself rather than to differences in pretrained checkpoints, external datasets, or training budget. Since this from-scratch setting is applied identically to every model, including the proposed EDC-Net, no method gains an advantage from additional external data, and all methods are therefore compared on an equal footing. For all methods, the input resolution was fixed at 640*×*640, the number of training epochs was set to 250, and the same data augmentation strategy, including Mosaic, HSV jittering, and random horizontal flipping, was used. For all CNN-based detectors, including SSD, Faster R-CNN, the YOLO-family detectors, Hyper-YOLO, and the proposed EDC-Net, the unified optimizer (SGD) and initial learning rate (0.01) listed in Table 2 were strictly applied. For RT-DETR, due to its Transformer-based architecture and distinct optimization behavior, its officially recommended optimizer (AdamW) and learning rate were adopted to ensure proper convergence and representative baseline performance. All models were evaluated under the same inference environment described in Table 1.

The specific code sources and model versions are as follows. SSD and Faster R-CNN were implemented using the MMDetection toolbox, where SSD adopted the SSD300 configuration with a VGG-16 backbone and Faster R-CNN adopted the ResNet-50-FPN configuration. For the YOLO-family detectors, the corresponding small-scale versions were used, including YOLOv5s, YOLOv8s, YOLOv10s, YOLO11s, YOLOv12s, and YOLOv13s. Among them, YOLOv5s, YOLOv8s, and YOLO11s were implemented within the Ultralytics framework, while YOLOv10s, YOLOv12s, and YOLOv13s were implemented based on their official repositories. Hyper-YOLO adopted the Hyper-YOLO-S version, and RT-DETR adopted the RT-DETR-R18 version with a ResNet-18 backbone. Both methods were implemented based on their official repositories.

### Analysis of experimental results

#### Comparative experiments

To verify the effectiveness of the proposed Edge-Aware Enhancement Module (EAEM), this study conducts comparative experiments on the VisDrone2019 dataset. Specifically, this study replaces the feature extraction module of the baseline model with advanced structures OREPA^36^, WDBB^37^, MogaBlock^38^, and FADC^39^. The experimental results are shown in Table 3, with the best values in bold.

**Table 3:** Performance comparison of feature extraction blocks.

Method	P	R	mAP@50	mAP@50:95	Paras	Size	GFLOPs	FPS	Latency
	(%)	(%)	(%)	(%)	(M)	(MB)			(ms)
Baseline	50.4	37.6	38.6	23.1	9.4	18.3	21.3	185.8	5.4
Baseline+OREPA	50.0	37.9	38.7	23.1	9.4	18.3	21.3	176.1	5.7
Baseline+WDBB	50.1	37.8	38.8	23.2	9.3	18.1	21.3	172.4	5.8
Baseline+MogaBlock	48.5	36.9	37.7	22.3	**9.2**	18.0	22.2	89.6	11.2
Baseline+FADC	**51.1**	38.4	39.5	23.7	9.5	18.4	21.1	111.0	9.0
Baseline+EAEM(Ours)	50.6	**38.8**	**39.6**	**23.8**	**9.2**	**17.9**	21.5	173.0	5.8

The results show that the EAEM achieves the best overall performance. EAEM reaches an mAP@50 of *39.6%* and the highest recall of *38.8%* without adding parameters, proving its advantage in capturing small objects. OREPA and WDBB show only slight improvements. MogaBlock reduces the model size slightly, but its mAP@50 drops to *37.7%*. FADC achieves the highest precision due to its frequency-adaptive mechanism, but it increases the parameter count and model size. Compared to FADC, the EAEM has a higher mAP@50 and a 0.5 MB smaller model size. This indicates that the EAEM can efficiently enhance feature representation without the need for extra parameters.

To further verify the robustness and cross-scenario adaptability of the proposed EAEM, supplementary experiments are conducted on the UAVDT dataset, which contains diverse UAV scenarios with variations in weather, flight altitudes, and camera viewpoints. As shown in Table 4, integrating EAEM into the baseline YOLO11s improves detection performance across VisDrone, TinyPerson, and UAVDT datasets. Specifically, mAP@50 is improved from 38.6% to 39.6% on VisDrone, from 17.5% to 18.7% on TinyPerson, and from 35.2% to 36.1% on UAVDT. These gains suggest that the edge cues extracted by EAEM are robust to scale variation, noise textures, and complex background edges, thanks to the dual-branch design and hierarchical embedding

**Table 4:** Cross-scenario performance of EAEM on different datasets.

Dataset	Model	mAP@50/%
VisDrone	YOLO11s	38.6
	YOLO11s+EAEM	**39.6**
TinyPerson	YOLO11s	17.5
	YOLO11s+EAEM	**18.7**
UAVDT	YOLO11s	35.2
	YOLO11s+EAEM	**36.1**

In order to assess the performance of the proposed EDC-Net, this study compares it with mainstream object detection algorithms. These algorithms include the two-stage Faster R-CNN, the one-stage SSD, multiple iterations of the YOLO series (YOLOv5s, YOLOv8s, YOLOv10s^40^, YOLO11s, YOLOv12s^41^, YOLOv13s^42^), as well as the hypergraph computation-based Hyper-YOLO^43^ and the Transformer-based real-time detector RT-DETR^44^.

**Table 5:** Comparative experiments on the VisDrone2019 dataset.

Method	P	R	mAP@50	mAP@50:95	Paras	Size	GFLOPs	FPS	Latency
	(%)	(%)	(%)	(%)	(M)	(MB)			(ms)
SSD	20.5	33.9	24.1	11.9	26.3	95.2	62.8	35.4	28.2
Faster R-CNN	45.4	35.6	33.2	17.3	41.2	108.5	370.2	15.2	65.7
YOLOv5s	48.2	37.5	38.0	22.7	7.8	15.2	19.0	226.7	4.4
YOLOv8s	50.1	37.4	38.4	22.9	9.8	19.0	23.6	238.6	4.2
YOLOv10s	50.2	37.3	38.5	23.0	7.2	15.8	21.4	190.6	5.3
YOLOv11s	50.4	37.6	38.6	23.1	9.4	18.3	21.3	185.8	5.4
YOLOv12s	47.8	37.8	38.3	22.8	9.1	17.8	19.6	106.2	9.4
YOLOv13s	48.4	37.3	37.5	22.2	9.0	17.7	20.4	67.7	14.8
Hyper-YOLO	51.6	39.3	40.5	24.6	13.5	26.2	33.8	162.5	6.2
MFA-YOLO^45^	-	-	42.6	-	9.6	-	34.0	85	-
DDSC-YOLO^46^	52.9	40.3	42.2	25.5	**5.0**	-	-	-	-
LMF-UAV-1^47^	52.0	39.7	41.1	24.6	14.7	-	61.8	-	-
CPDD-YOLOv8^48^	51.7	41.7	41.0	23.5	20.6	-	141.9	22	-
RT-DETR	**54.3**	40.5	42.7	25.9	19.9	77.0	57.0	62.3	16.1
EDC-Net(Ours)	53.8	**41.7**	**43.1**	**26.1**	6.6	**13.1**	22.5	172.4	5.8

Table 5 presents the experimental comparisons across the VisDrone2019 dataset. The results reveal that SSD has a low detection accuracy, and only an mAP@50 of *24.1%* is obtained. As for Faster R-CNN, the large number of parameters (41.2 M) makes it difficult to meet the requirements of edge deployment. In contrast, EDC-Net exhibits the best comprehensive performance, and outperforms other compared methods in the two main metrics, mAP@50 and mAP@50:95. Relative to the original YOLO11s, gains of *4.5%* and *3.0%* are attained for the mAP@50 and mAP@50:95 metrics, respectively. EDC-Net also obtains the best recall (*41.7%*), with improvements of *2.4%* and *1.2%* over Hyper-YOLO and RT-DETR respectively, reducing the missed detection rate of small objects. While RT-DETR achieves better precision (*54.3%*), it introduces greater model complexity. Its parameter count (19.9 M) and model size (77.0 MB) are 3.0 and 5.9 times those of the proposed method, respectively, restricting its application on resource-limited devices. By contrast, EDC-Net attains an mAP@50 value of *43.1%* with a much smaller parameter count of only 6.6 M and model size of 13.1 MB. Although EDC-Net has slightly higher GFLOPs than the baseline YOLO11s, the additional computational cost remains acceptable. These results indicate that the proposed method improves detection accuracy while maintaining a reasonable balance among model complexity, computational cost, and inference efficiency. To further demonstrate the competitiveness of EDC-Net, several recently developed UAV small-object detection models, including MFA-YOLO, DDSC-YOLO, LMF-UAV, and CPDD-YOLOv8, are additionally included for comparison. Among all compared methods, EDC-Net achieves the highest mAP@50 and mAP@50:95, reaching 43.1% and 26.1%, respectively. Although DDSC-YOLO has fewer parameters, EDC-Net obtains higher detection accuracy. Compared with CPDD-YOLOv8, EDC-Net also achieves better mAP@50 and mAP@50:95 with fewer parameters and lower GFLOPs, further demonstrating its effectiveness in UAV small-object detection.

**Fig. 6:**
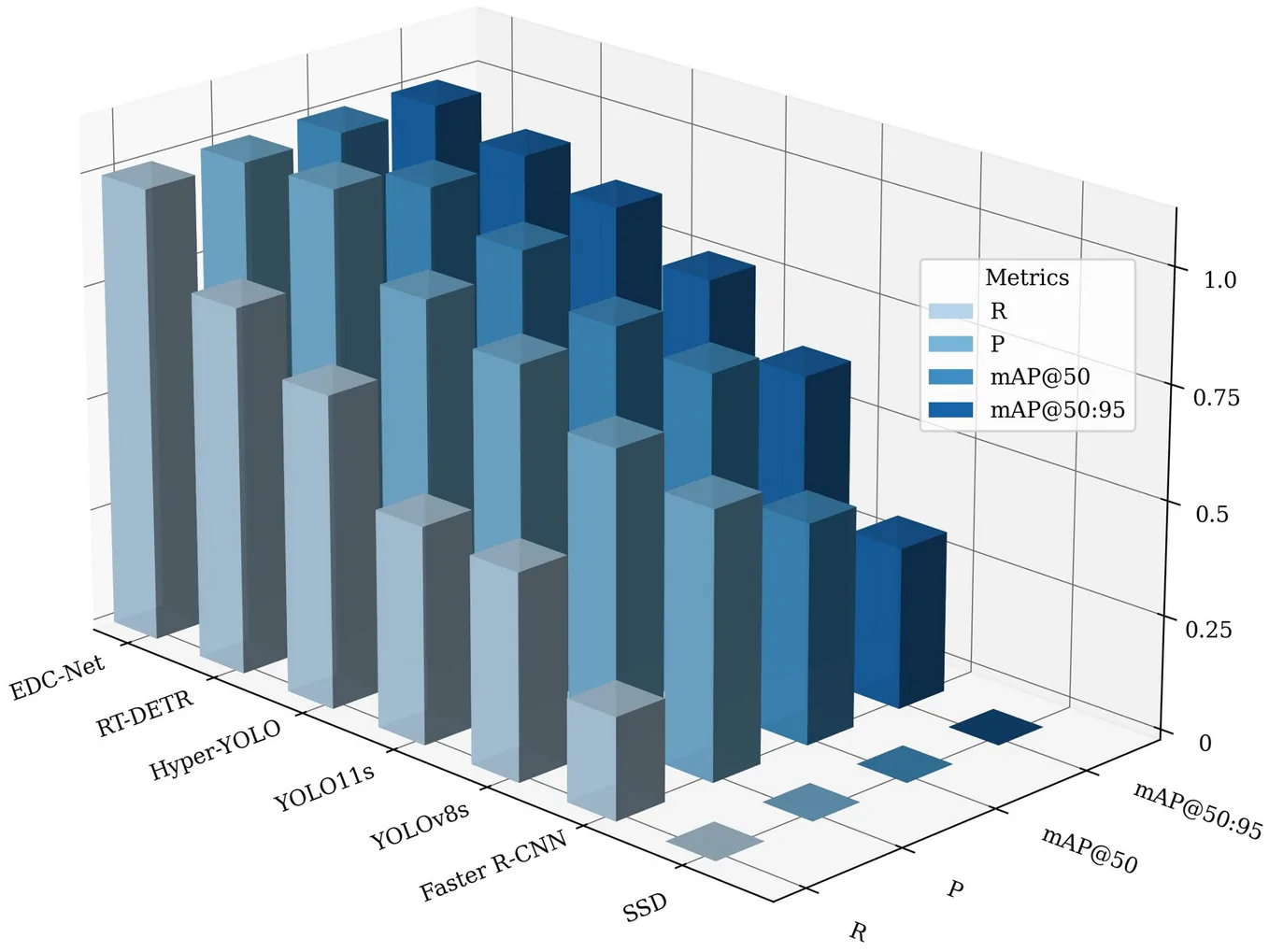
Visualization of the main comparative experiments on the VisDrone2019 dataset.

Figure 6 compares the key performance metrics of various models on the VisDrone2019 dataset, further confirming the advantages of the proposed method in handling complex aerial scenarios.

To verify the generalization ability of EDC-Net, this paper conducts comparative experiments on the TinyPerson dataset. The average target size in the TinyPerson dataset is very small (around 18 pixels), making the detection task very difficult.

**Table 6:** Comparative experiments on the TinyPerson dataset.

Method	P	R	mAP@50	mAP@50:95	Paras	Size	GFLOPs	FPS	Latency
	(%)	(%)	(%)	(%)	(M)	(MB)			(ms)
SSD	13.5	8.2	7.3	1.1	26.3	95.1	62.8	35.5	28.2
Faster R-CNN	17.3	9.8	9.2	2.6	41.2	108.3	370.2	15.3	65.2
YOLOv5s	35.6	18.7	16.7	5.5	7.8	15.2	19.0	225.1	4.4
YOLOv8s	35.3	18.6	16.9	5.7	9.8	19.0	23.6	246.2	4.1
YOLOv10s	33.9	18.8	17.2	5.8	7.2	15.8	21.4	182.1	5.5
YOLOv11s	36.1	18.9	17.5	5.9	9.4	18.3	21.3	186.2	5.4
YOLOv12s	34.2	18.1	15.6	5.1	9.1	17.8	19.6	105.6	9.5
YOLOv13s	31.4	17.0	14.7	4.8	9.0	17.7	20.4	72.2	13.9
Hyper-YOLO	35.9	18.4	16.8	5.6	13.5	26.2	33.8	165.8	6.0
MEU-YOLOv5s^49^	**37.1**	23.7	20.4	6.8	-	-	36.9	-	-
PS-YOLO-s^50^	33.1	18.7	17.7	6.4	-	-	-	-	-
PS-YOLO-X^50^	36.9	20.1	19.6	7.1	-	-	-	-	-
RT-DETR	36.7	23.6	20.2	6.4	19.9	77.0	56.9	63.3	15.8
EDC-Net(Ours)	37.0	**25.7**	**21.5**	**7.1**	**6.6**	**13.2**	22.5	170.1	5.9

Table 6 presents the experimental results. Confronted with the challenge of extremely small object detection, EDC-Net still achieves the optimal performance, significantly outperforming SSD, Faster R-CNN, and YOLO series models. Compared with the baseline model YOLO11s, EDC-Net increases the mAP@50 by 4.0% to 21.5%. Furthermore, it greatly improves the recall by 6.8% to 25.7%, alleviating the missed detection problem of tiny objects. Compared with RT-DETR, our method not only gets a 1.3% higher mAP@50 but also reduces parameters by about 67% and model size by 83%. Although its GFLOPs are slightly higher than those of the baseline YOLO11s, the model reduces the parameter count and model size while keeping the additional inference overhead within a reasonable range. This suggests that the improved detection performance is achieved without introducing excessive computational burden. Beyond the general-purpose detectors, EDC-Net is further compared with the recently developed small-object detection models MEU-YOLOv5s and PS-YOLO variants. EDC-Net achieves the highest mAP@50 of 21.5% among the compared methods. Compared with the much larger PS-YOLO-X, EDC-Net obtains a higher mAP@50 and a comparable mAP@50:95 with a smaller parameter count, further indicating its effectiveness on the TinyPerson dataset.

**Fig. 7:**
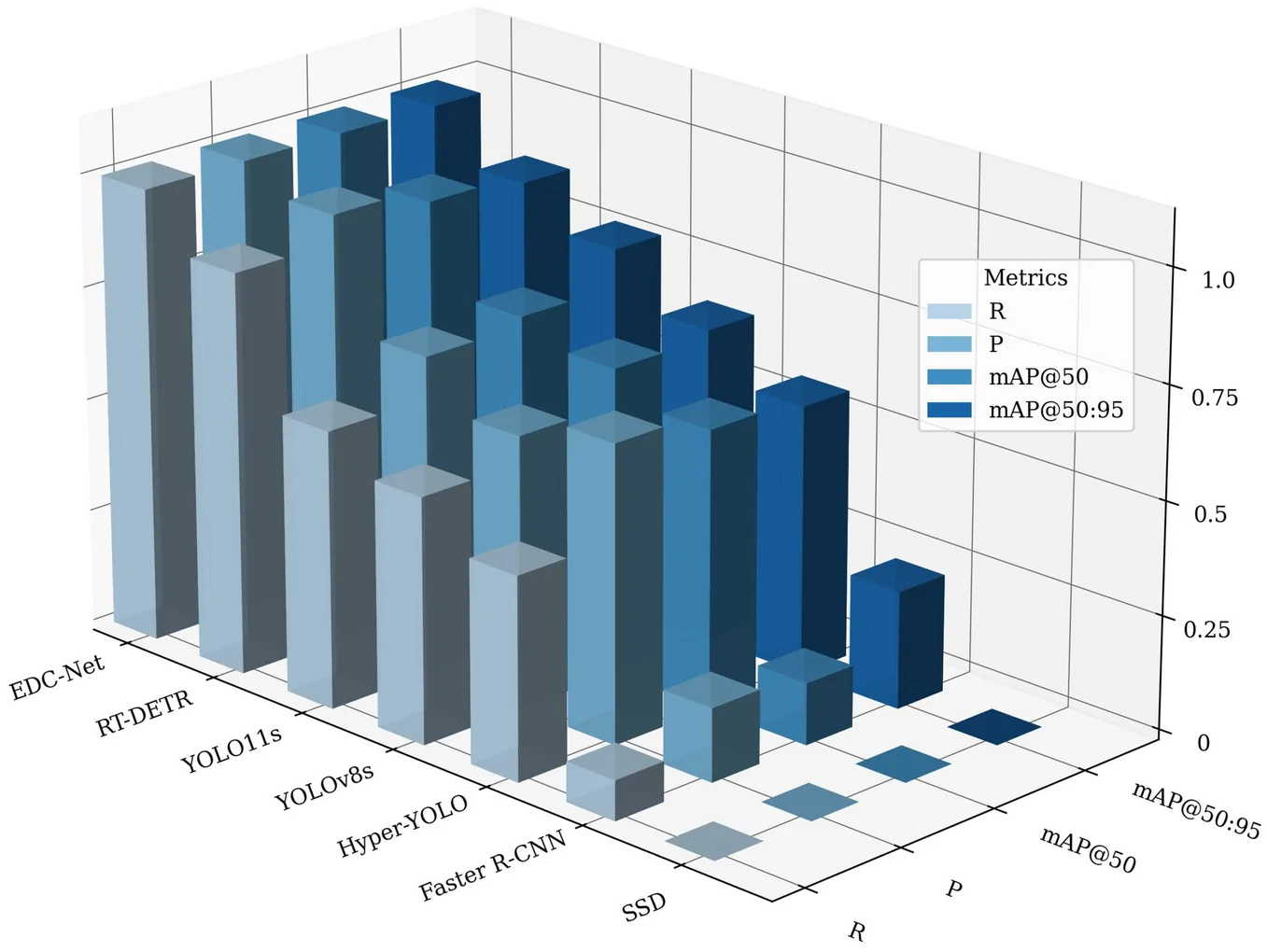
Visualization of the main comparative experiments on the TinyPerson dataset.

As shown in Fig. 7, the key metrics on the TinyPerson dataset further demonstrate the robustness of our method in detecting tiny objects.

**Table 7:** AP comparison for objects of different scales on the VisDrone2019 dataset.

Model	AP$_{S}$ (%)	AP$_{M}$ (%)	AP$_{L}$ (%)
YOLO11s	12.8	32.6	40.8
EDC-Net(Ours)	**16.2**	**35.4**	**42.9**

To further analyze the performance of EDC-Net on objects of different scales, AP$_{S}$, AP$_{M}$, and AP$_{L}$ are reported on the VisDrone2019 dataset, as shown in Table 7. Compared with YOLO11s, EDC-Net improves AP$_{S}$, AP$_{M}$, and AP$_{L}$ from 12.8%, 32.6%, and 40.8% to 16.2%, 35.4%, and 42.9%, respectively. The largest gain is achieved on small objects, with an improvement of 3.4 percentage points. This result further indicates that the edge cues enhanced by EAEM, the multi-scale adaptive context modeling of MACM, and the topology-constrained sampling in DSAH are effective for improving small-object detection in complex UAV aerial scenes. The gains on medium and large objects also show that the proposed method maintains stable performance across different object scales.

Table 8 presents a comparison of the detection performance between YOLO11s and EDC-Net in scenarios with different densities and scales. The experimental results show that the improved algorithm has significant advantages on both the VisDrone2019 and TinyPerson datasets, which fully verifies its generalization ability in different scenarios.

**Table 8:** Comparison between the baseline model and the improved model.

Dataset	Model	P/%	R/%	mAP@50/%	mAP@50:95/%
VisDrone	YOLO11s	50.4	37.6	38.6	23.1
	EDC-Net	**53.8**	**41.7**	**43.1**	**26.1**
TinyPerson	YOLO11s	36.1	18.9	17.5	5.9
	EDC-Net	**37.0**	**25.7**	**21.5**	**7.1**

#### Ablation experiments

To verify the effectiveness of each improved module, ablation experiments are performed on both the VisDrone2019 and TinyPerson datasets. All experiments are conducted with the same hyperparameters and in the same environments for fair comparison, and the results are provided in Tables 9 and 10.

Table 9 details the ablation study conducted on VisDrone2019. Compared with the baseline YOLO11s, adding the EAEM module improves mAP@50 by *1.0%* and slightly reduces the parameter count. This proves that the module can effectively extract blurred edge features. On this basis, replacing the original C2PSA with the redesigned MACM further increases mAP@50 and mAP@50:95 to *40.2%* and *24.1%*, respectively. This shows that MACM is capable of fusing multi-scale contextual information and boosting the model’s capability to suppress background noise. With the first two modules integrated, using the improved DSAH detection head not only reduces the parameter count by about *28.3%* but also significantly improves precision. Specifically, mAP@50 improves by *2.9%*, and mAP@50:95 gains *2.0%*. The relatively large gain brought by DSAH indicates that geometry-aware localization is an important factor for improving detection accuracy in dense UAV scenes. However, EAEM and MACM remain essential components: their combination still improves mAP@50 from 38.6% to 40.2% by enhancing weak contour cues and multi-scale contextual representation. More importantly, when combined with DSAH, they further improve mAP@50 from 41.1% to 43.1% and mAP@50:95 from 24.6% to 26.1%, showing that the three modules play complementary roles at different stages of feature extraction, context modeling, and localization refinement. After integrating all three modules, the improved model achieves *43.1%* mAP@50, *26.1%* mAP@50:95, *53.8%* precision, and *41.7%* recall. Compared with the baseline model, these metrics are improved by *4.5%*, *3.0%*, *3.4%*, and *4.1%*, respectively. Meanwhile, the parameter count and model size are reduced by *29.8%* and *28.4%*, respectively. These results demonstrate that the proposed algorithm maintains good detection performance while effectively reducing model size and storage cost.

**Table 9:** Ablation experiments on the VisDrone2019 dataset.

Model	EAEM	MACM	DSAH	P	R	mAP@50	mAP@50:95	Paras	Size	GFLOPs	FPS	Latency
				(%)	(%)	(%)	(%)	(M)	(MB)			(ms)
YOLO11s				50.4	37.6	38.6	23.1	9.4	18.3	21.3	185.8	5.4
	*\checkmark*			50.6	38.8	39.6	23.8	9.2	17.9	21.5	173.0	5.8
		*\checkmark*		50.3	38.7	39.9	24.0	9.4	18.3	21.3	190.7	5.2
			*\checkmark*	**53.9**	40.8	41.1	24.6	6.8	13.5	22.3	218.9	4.1
	*\checkmark*	*\checkmark*		50.3	39.4	40.2	24.1	9.2	17.9	21.5	163.4	6.1
	*\checkmark*	*\checkmark*	*\checkmark*	53.8	**41.7**	**43.1**	**26.1**	**6.6**	**13.1**	22.5	172.4	5.8

**Table 10:** Ablation experiments on the TinyPerson dataset.

Model	EAEM	MACM	DSAH	P	R	mAP@50	mAP@50:95	Paras	Size	GFLOPs	FPS	Latency
				(%)	(%)	(%)	(%)	(M)	(MB)			(ms)
YOLO11s				36.1	18.9	17.5	5.9	9.4	18.3	21.3	186.2	5.4
	*\checkmark*			36.3	19.9	18.7	6.0	9.2	17.9	21.5	170.2	5.9
		*\checkmark*		36.0	20.2	18.4	5.9	9.4	18.4	21.3	173.9	5.8
			*\checkmark*	36.2	24.0	19.7	6.5	6.8	13.5	22.2	210.6	4.8
	*\checkmark*	*\checkmark*		35.9	19.5	18.9	6.1	9.2	18.0	21.5	151.7	6.6
	*\checkmark*	*\checkmark*	*\checkmark*	**37.0**	**25.7**	**21.5**	**7.1**	**6.6**	**13.2**	22.5	170.1	5.9

In addition, Table 10 documents the ablation outcomes on the TinyPerson dataset, further verifying the generalization capacity of the presented algorithm. The final improved model attains an mAP@50 value of *21.5%* on this dataset, an improvement of *4.0%* over the baseline. The increase in recall from *18.9%* to *25.7%* (an increase of *6.8%*) proves that the proposed improvement strategy maintains detection stability even when facing a scarcity of features for tiny objects.

**Table 11:** Ablation experiments of the MACM module.

Model	DyT	TSSA	Mona	P	R	mAP@50	mAP@50:95	Paras	Size	GFLOPs	FPS	Latency
				(%)	(%)	(%)	(%)	(M)	(MB)			(ms)
YOLO11s				**50.4**	37.6	38.6	23.1	9.4	18.3	21.3	185.8	5.4
	*\checkmark*			49.6	38.2	38.9	23.4	9.4	18.3	21.3	205.8	4.9
		*\checkmark*		50.0	38.1	39.0	23.4	**9.3**	**18.2**	21.3	169.5	5.9
			*\checkmark*	50.3	38.3	39.4	23.7	9.5	18.5	21.4	164.6	6.1
	*\checkmark*	*\checkmark*		49.9	38.5	39.2	23.5	**9.3**	18.3	21.3	162.9	6.1
+MACM	*\checkmark*	*\checkmark*	*\checkmark*	50.3	**38.7**	**39.9**	**24.0**	9.4	18.3	21.3	190.7	5.2

Table 11 shows the internal ablation results of MACM on the VisDrone dataset. The results demonstrate that each component improves the detection performance of the baseline model. Specifically, DyT dynamically suppresses background noise to optimize the feature signal-to-noise ratio, which increases the mAP@50 by *0.3%*. TSSA uses a token statistical mechanism to capture long-range global dependencies. This overcomes the local constraints of CNNs and raises the mAP@50 to *39.0%*. Using multi-kernel parallel convolutional branches, Mona refines the perception of multi-scale objects and achieves the best single-component performance of *39.4%*. Stepwise integration experiments show the synergy among these components. When fully integrated, MACM attains an mAP@50 value of *39.9%* and a recall rate of *38.7%*. Compared to the baseline, these represent improvements of *1.3%* and *1.1%*, respectively. Importantly, the parameter count remains similar to the baseline despite the precision improvements. This shows that the performance gain comes from optimizing internal feature interactions rather than simply stacking parameters, which fundamentally improves feature fusion.

**Table 12:** Comparison between MACM and mainstream attention mechanisms.

Method	P/%	R/%	mAP@50/%	mAP@50:95/%	Paras/M	Size/MB
Baseline	50.4	37.6	38.6	23.1	9.4	18.3
Baseline+SimAM	49.7	38.0	38.9	23.3	9.4	18.3
Baseline+EMA	50.1	38.1	38.9	23.4	9.5	18.4
Baseline+SE	**50.5**	37.8	39.4	23.7	9.4	18.4
Baseline+ECA	50.3	38.2	39.2	23.3	9.4	18.3
Baseline+CBAM	48.6	38.1	38.8	23.5	9.4	18.4
Baseline+MACM(Ours)	50.3	**38.7**	**39.9**	**24.0**	9.4	18.3

To further demonstrate the effectiveness of the integrated design of MACM, representative attention mechanisms and multi-scale convolution modules are compared with MACM on the VisDrone2019 dataset. For fairness, only the C2PSA module in YOLO11s is replaced in all experiments. As shown in Tables 12 and 13, MACM achieves the best recall, mAP@50, and mAP@50:95 among both groups, reaching 38.7%, 39.9%, and 24.0%, respectively, without increasing the parameter count or model size. These results indicate that the progressive integration of noise suppression, global context modeling, and multi-scale local refinement is more effective than isolated attention mechanisms or multi-scale convolution modules for UAV small object detection.

**Table 13:** Comparison between MACM and multi-scale convolution modules.

Method	P/%	R/%	mAP@50/%	mAP@50:95/%	Paras/M	Size/MB
Baseline	**50.4**	37.6	38.6	23.1	9.4	18.3
Baseline+PKI	49.0	38.6	39.1	23.4	9.3	18.3
Baseline+FFCM	**50.4**	37.7	38.9	23.3	9.6	18.8
Baseline+ODConv	48.6	38.4	38.8	23.3	9.5	18.4
Baseline+MBRConv	50.1	38.5	39.4	23.6	9.7	19.3
Baseline+MACM(Ours)	50.3	**38.7**	**39.9**	**24.0**	9.4	18.3

## Visualization analysis

**Fig. 8:**
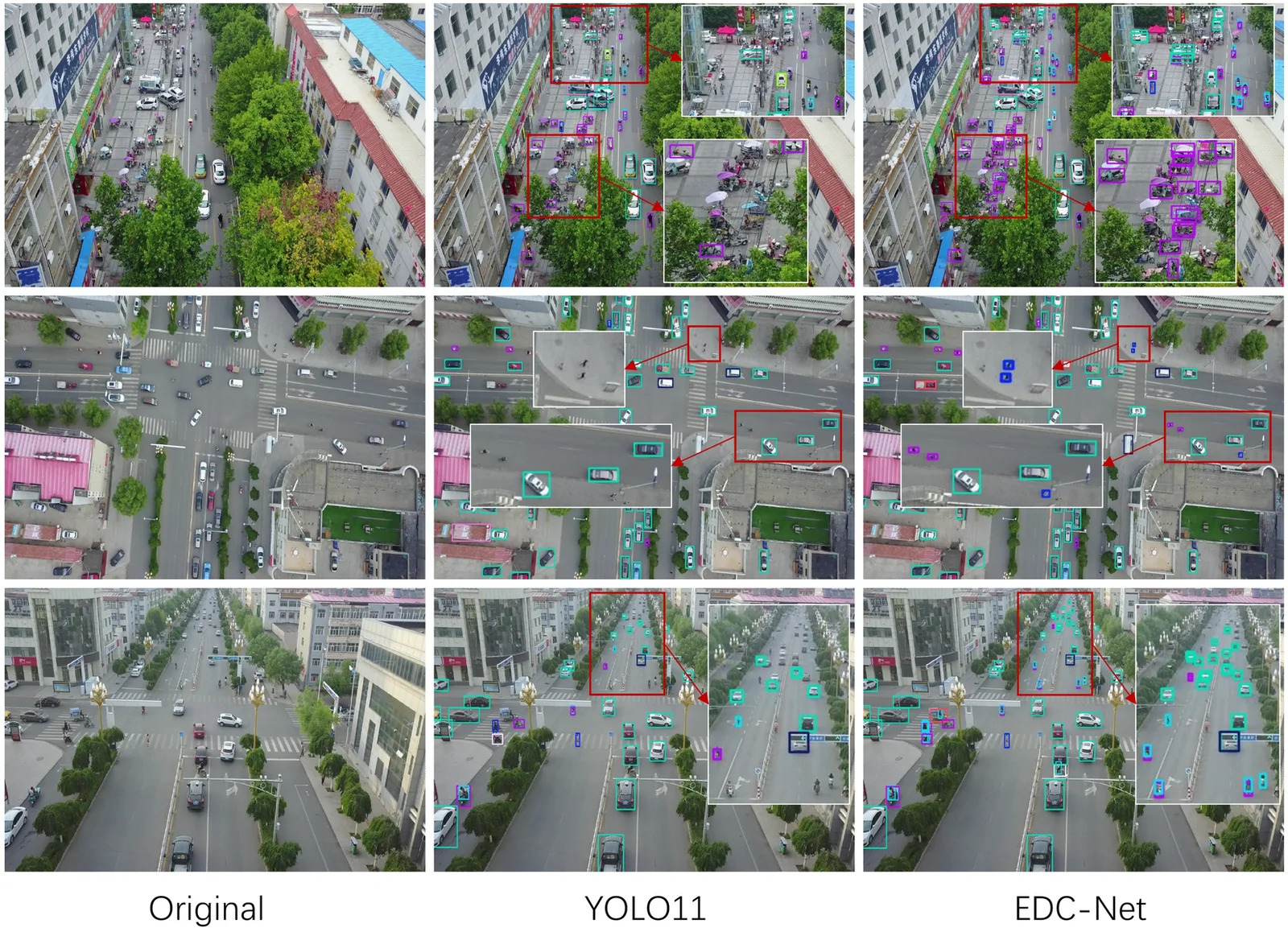
Comparison of detection results on the VisDrone2019 dataset.

In order to visually illustrate the detection performance of the improved network, we present detection results and heatmaps to compare the performance before and after the improvement. Fig. 8 presents a comparison of detection results from YOLO11 and EDC-Net on the VisDrone2019 dataset.

Here we choose three typical scenarios for analysis: the first row represents a dense object distribution scenario, the second row represents a high-altitude overhead scenario, and the third row represents a scale imbalance scenario. In the dense object distribution scenario, EDC-Net can recognize distant tiny pedestrians, whereas YOLO11 exhibits missed detections. This is because EDC-Net, by enhancing boundary feature representation, effectively alleviates issues such as severe occlusion and blurred boundaries among small objects, resulting in more complete and precise bounding boxes. In the high-altitude overhead scenario, YOLO11 struggles to detect tiny objects with low background contrast, while EDC-Net enhances the discriminability of small objects by introducing a more effective multi-scale adaptive contextual structure. In the scale imbalance scenario, EDC-Net accurately detects distantly and densely distributed vehicles and pedestrians, exhibiting detection stability superior to that of YOLO11.

**Fig. 9:**
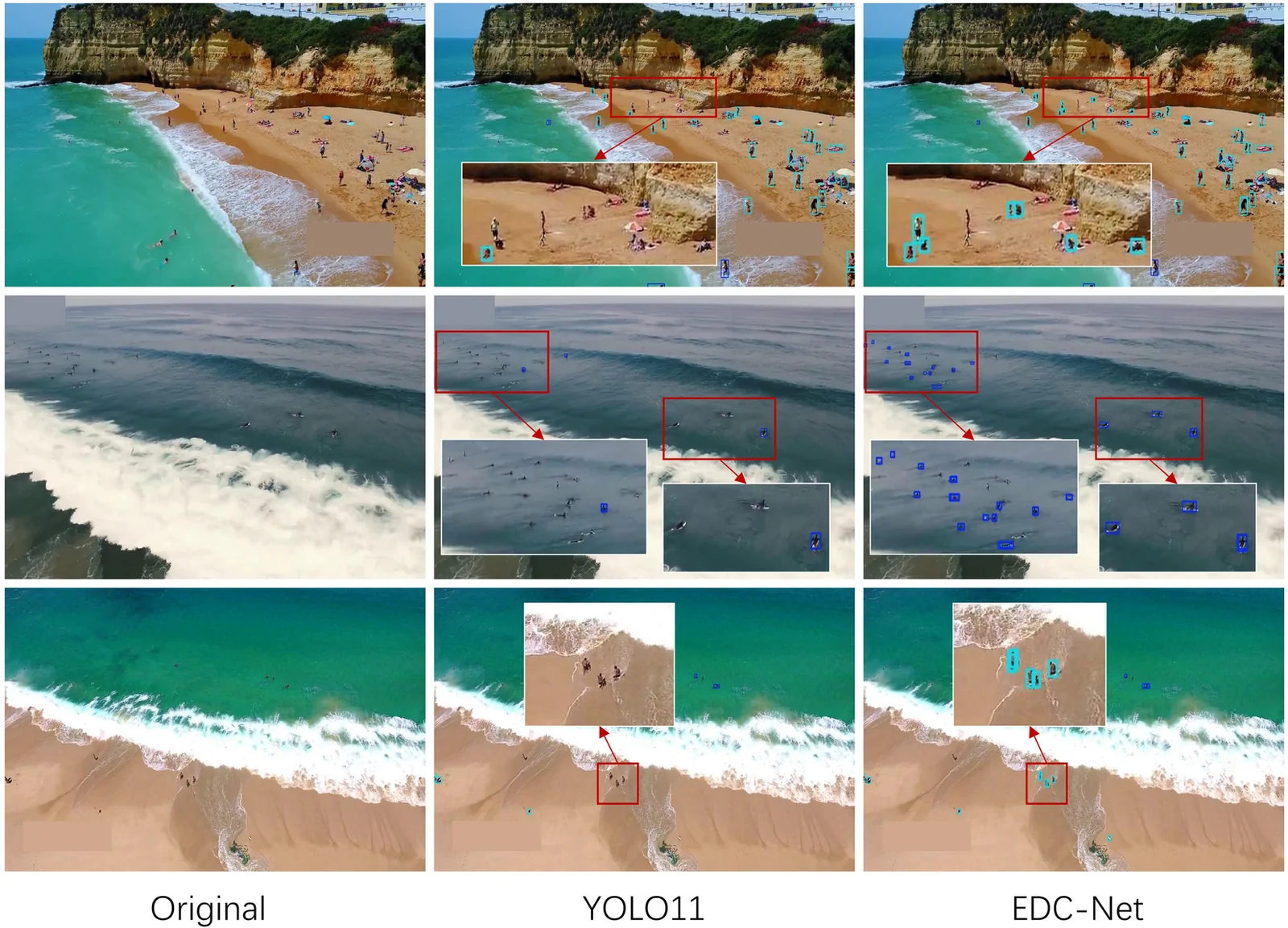
Comparison of detection results on the TinyPerson dataset.

Figure 9 is the visual comparison of typical cases on the TinyPerson dataset. EDC-Net exhibits a more acute detection ability for tiny objects. It can still capture faint features in the critical sea surface case and recognize the tiny objects under sea surface disturbance.

**Fig. 10:**
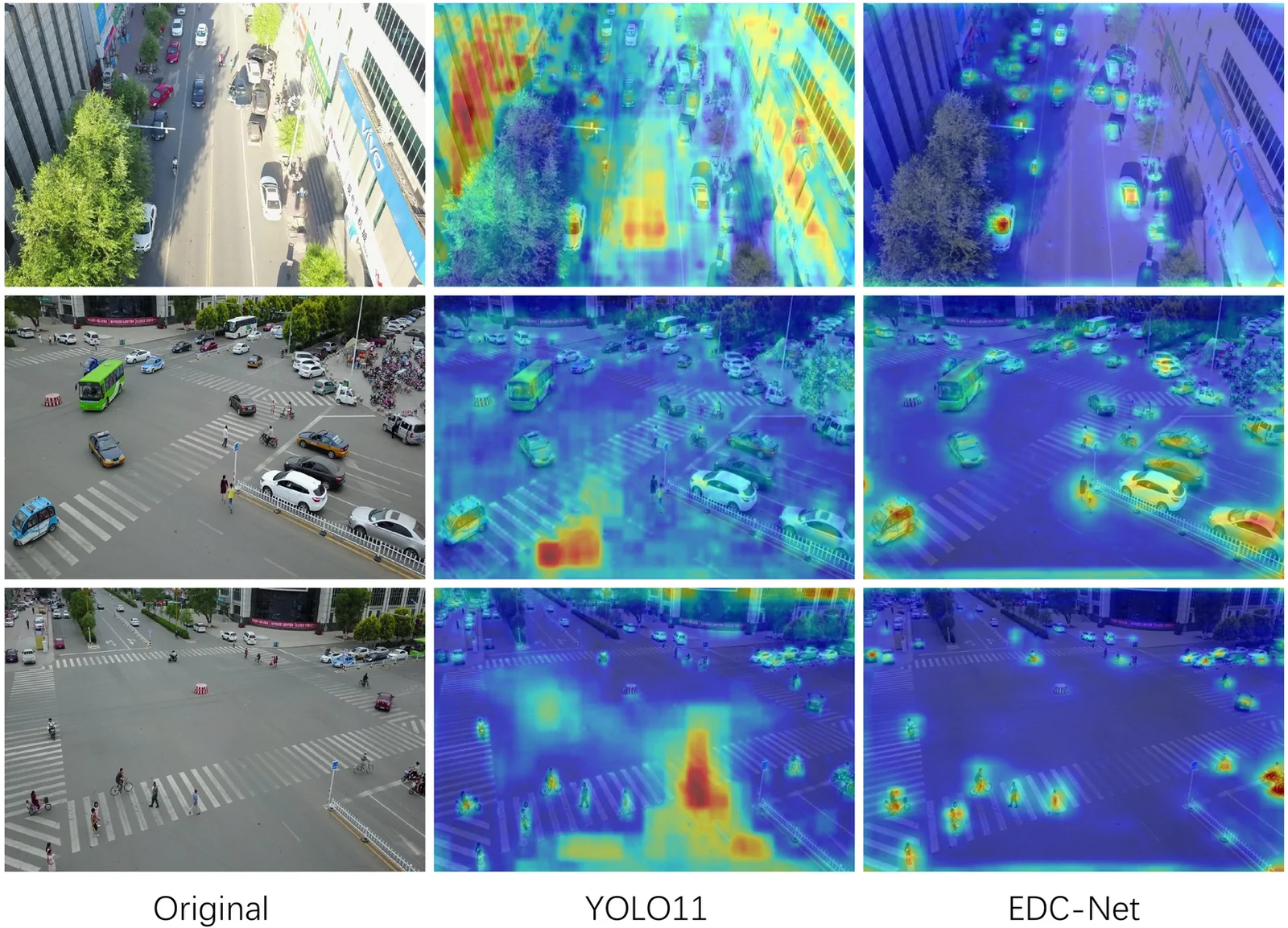
Heatmap comparison between YOLO11 and EDC-Net.

Furthermore, the heatmap comparison results in Fig. 10 reveal that whether in vehicle-dense intersections or tree-occluded street backgrounds, EDC-Net exhibits stronger feature focusing capabilities. Compared to the baseline model, its high-response regions not only precisely cover the main bodies of the objects but also successfully activate multiple small-scale objects missed by YOLO11. EDC-Net shows better robustness and localization accuracy in many complex scenarios. This proves that the improved methods work well.

## Conclusion

This paper proposes a UAV aerial small object detection method called EDC-Net, aimed at addressing the problem of poor accuracy in the detection of small objects. The model integrates three core components: the Edge-Aware Enhancement Module (EAEM), the Multi-scale Adaptive Context Module (MACM), and the Dynamic Structural Alignment Head (DSAH). By coordinating edge-aware enhancement in the backbone, adaptive context modeling in deep features, and dynamic structural alignment in the detection head, EDC-Net improves feature discriminability and localization accuracy for UAV small object detection under complex backgrounds and scale variations. In particular, EAEM uses explicit gradient information to guide the network in focusing on the contour of tiny objects, and effectively compensates for the missing texture details in deep features. By combining dynamic non-linear activation, multi-scale visual perception, and statistical self-attention, the MACM refines feature representation for multi-scale objects while also suppressing complex background interference. The DSAH boosts the model’s adaptive modeling capacity for object geometric structures via dynamic snake convolutions, improving the model’s localization accuracy. Extensive experimental results on the VisDrone2019 and TinyPerson datasets show that the proposed method has great advantages over existing methods in small object detection tasks in terms of accuracy and robustness.

Despite achieving performance improvements in feature extraction and detection head design, EDC-Net still faces limitations. Specifically, targeted optimization for the neck network has not yet been conducted in the current work. Furthermore, considering the resource-constrained nature of UAV platforms, network efficiency remains a critical challenge. Future work will address the design of efficient necks and introduce model pruning and knowledge distillation to improve the real-time performance of the model while maintaining accuracy, so as to meet the deployment requirements of edge devices.

## Data Availability

All datasets used in this study are publicly available. The VisDrone2019 dataset can be downloaded from the offfcial repository maintained by the AISKYEYE team, Tianjin University, at https://github.com/VisDrone/VisDrone-Dataset. The TinyPerson dataset is available at https://github.com/ucas-vg/TinyBenchmark. The UAVDT dataset is available at its offfcial project page, https://sites.google.com/view/grli-uavdt/. The core source code of the proposed modules and the corresponding training conffgurations used in the experiments are publicly available at https://github.com/only-yyang/EDC-Net.
